# 

**Published:** 1856-10-01

**Authors:** 


### CONTENTS.

PAGE

Psychological Quarterly Retrospect . . . . . . . . . lxiii—xc

PART I.—ORIGINAL COMMUNICATIONS.

1. On Monomania . . . . . . . . . . . . . . . . . . . 502
2. Woman in her Social Relations, Past and Present . . . . . . . . 521
3. On the Physiological and Psychological Phenomena of Dreams and Apparitions . . . . . . . . . . . . . . . . . . . . 544
4. Leaves from the Diary of a Patient confined in Hanwell Asylum . . 562
5. On the Connexion between Morbid Physical and Moral Phenomena . 572
6. William Dove . . . . . . . . . . . . . . . . . . . 584
7. Triune Man.—The Inaugural Discourse delivered at the opening of the last Session of the London Hospital Medical College . . . 594
8. Psychology of Malebranche . . . . . . . . . . . . . . . 608

PART II.

1. The Statistics of Insanity, and Idiocy and Cretinism.—Reports of the International Statistical Congress . . . . . . . . . . . . 628
2. The District Hospitals for the Insane in Ireland.—Superannuations . 642

PART III.—REVIEWS.

1. Nomos: an Attempt to Demonstrate a Central Physical Law in Nature 644
2. Obscure Nervous Diseases popularly explained . . . . . . . . . 644

PART IV.—JUDICIAL DEPARTMENT.

1. Annual Meeting of the Association of Medical Officers of Asylums and Hospitals for the Insane . . . . . . . . . . . . . . . 646
2. The Confession of Dove . . . . . . . . . . . . . . . . 650
3. Statistics of Crime . . . . . . . . . . . . . . . . . . 659

Index to Volume IX.

